# Biokinetics, dosimetry, and radiation risk in infants after ^99m^Tc-MAG3 scans

**DOI:** 10.1186/s13550-017-0356-2

**Published:** 2018-02-02

**Authors:** J. Soares Machado, J. Tran-Gia, S. Schlögl, A. K. Buck, M. Lassmann

**Affiliations:** Department of Nuclear Medicine, University Hospital Würzburg, Oberdürrbacher Str. 6, 97080 Würzburg, Germany

**Keywords:** Pediatric patients, Dosimetry, ^99m^Tc-MAG3, Biokinetics, Absorbed dose, Risk assessment

## Abstract

**Background:**

Renal scans are among the most frequent exams performed on infants and toddlers. Due to the young age, this patient group can be classified as a high-risk group with a higher probability for developing stochastic radiation effects compared to adults. As there are only limited data on biokinetics and dosimetry in this patient group, the aim of this study was to reassess the dosimetry and the associated radiation risk for infants undergoing ^99m^Tc-MAG3 renal scans based on a retrospective analysis of existing patient data.

Consecutive data were collected from 20 patients younger than 20 months (14 males; 6 females) with normal renal function undergoing ^99m^Tc-MAG3 scans. To estimate the patient-specific organ activity, a retrospective calibration was performed based on a set of two 3D-printed infant kidneys filled with known activities. Both phantoms were scanned at different positions along the anteroposterior axis inside a water phantom, providing depth- and size-dependent attenuation correction factors for planar imaging. Time-activity curves were determined by drawing kidney, bladder, and whole-body regions-of-interest for each patient, and subsequently applying the calibration factor for conversion of counts to activity. Patient-specific time-integrated activity coefficients were obtained by integrating the organ-specific time-activity curves. Absorbed and effective dose coefficients for each patient were assessed with OLINDA/EXM for the provided newborn and 1-year-old model. The risk estimation was performed individually for each of the 20 patients with the NCI Radiation Risk Assessment Tool.

**Results:**

The mean age of the patients was 7.0 ± 4.5 months, with a weight between 5 and 12 kg and a body size between 60 and 89 cm. The injected activities ranged from 12 to 24 MBq of ^99m^Tc-MAG3. The patients’ organ-specific mean absorbed dose coefficients were 0.04 ± 0.03 mGy/MBq for the kidneys and 0.27 ± 0.24 mGy/MBq for the bladder. The mean effective dose coefficient was 0.02 ± 0.02 mSv/MBq. Based on the dosimetry results, an evaluation of the excess lifetime risk for the development of radiation-induced cancer showed that the group of *newborns* has a risk of 16.8 per 100,000 persons, which is about 12% higher in comparison with the *1-year-old* group with 14.7 per 100,000 persons (all values are given as mean plus/minus one standard deviation except otherwise specified).

**Conclusion:**

In this study, we retrospectively derived new data on biokinetics and dosimetry for infants with normal kidney function after undergoing renal scans with ^99m^Tc-MAG3. In addition, we analyzed the associated age- and gender-specific excess lifetime risk due to ionizing radiation. The radiation-associated stochastic risk increases with the organ doses, taking age- and gender-specific influences into account. Overall, the lifetime radiation risk associated with the ^99m^Tc-MAG3 scans is very low in comparison to the general population risk for developing cancer.

**Electronic supplementary material:**

The online version of this article (10.1186/s13550-017-0356-2) contains supplementary material, which is available to authorized users.

## Background

According to information from the American National Institute of Diabetes and Digestive and Kidney Diseases, there is a high incidence of kidney pathologies in infants [1]. Compared to adults, they have a ~20 times higher probability for developing defects in the urinary tract. Cases as urine reflux/blockage and infections are related with the infants’ immature urinary system and/or malformation by birth [1]. Nuclear medicine renography for pediatric patients is one of the standard non-invasive diagnostic methods with advantages such as a potential detection of diseases in early stages and information on physiology with a high sensitivity [2, 3]. The standard tracer for examining the latter is ^99m^Tc-MAG3 [2].

^99m^Tc-MAG3 scans are often indicated for renal function examinations in infants because of the minimum recommended age (1 month), the short physical half-life of ^99m^Tc (6.01 h), the high extraction rate of the radiopharmaceutical (60% in the first filtration), and the high kidney uptake (97%), providing a good image quality even for infants [2]. Therefore, renal scans with ^99m^Tc-MAG3 are among the most frequent urinary tract exams performed on infants and toddlers [3].

The basic standard protocol for ^99m^Tc-MAG3 renal scans performed for young children consists of a dynamic scan performed on a single-headed camera equipped with a low-energy collimator [2]. According to the European Association of Nuclear Medicine (EANM) dosage card, the recommended minimum injected activity is 15 MBq [4].

While there are no recommended absorbed dose limits, it is, based on the ALARA concept (“As Low As Reasonably Achievable”), highly recommended to always minimize the doses with regard to the image quality necessary for an accurate diagnosis [3]. It is also important to consider the risk from radiation exposure for this group of patients, as, due to the young age, this group could potentially be classified as a “high-risk group” with a higher probability for developing stochastic radiation effects compared to adults [3]. Cancer is a complex disease that depends on many factors such as age, gender, genetic predisposition, and lifestyle, and it can take years to develop [1, 3, 5, 6]. Therefore, an exposure to ionizing radiation at young ages will, most likely, increase the cancer risk [7]. According to information provided by BEIR VII (Committee on the Biological Effects of Ionizing Radiation) about risk estimation for exposure in childhood (studied cohort: the atomic bomb survivors and two case-control studies of thyroid cancer), the risk decreases with the age at the time of exposure [6, 7]. Based on these considerations, one can conclude that an accurate risk assessment is of particular importance for pediatric patients to minimize the risk of nuclear medicine imaging procedures [3]. However, there is few data on biokinetics, dosimetry, and risk estimation at low doses for pediatric patients. In a review by Eberlein et al., it was shown that, for ^99m^Tc-MAG3 renal scans, the biokinetic and dosimetry data were published 26 years ago, with only four data sets specific for children [8].

One of the quantities proposed by the International Commission on Radiological Protection (ICRP) used for assessing radiation risk is the effective dose [5, 9]. According to ICRP, it can be applied in diagnostic exams to estimate the health detriment for a general group of exposed individuals, without considering ages and gender. Based on the effective dose values, the risk levels of different procedures can be compared and optimized whenever it seems reasonable or necessary [5, 9]. Therefore, the ICRP formalism is not applicable for performing individual risk estimation of radiation-induced effects.

A newly developed tool for individual patient risk estimation is the Radiation Risk Assessment Tool (RadRAT) developed by the National Cancer Institute’s Division of Cancer Epidemiology and Genetics [10]. This online platform tool was developed based on BEIR VII from the National Academy concerning radiation health effects named Health Risks from Exposure to Low Levels of Ionizing Radiation. Utilizing the RadRAT calculator, it is possible to perform lifetime attributable risk (LAR) estimations of radiation-related cancer induction for low-level ionizing radiation with doses < 1 Gy for individuals based on their age, gender, year of exposure, uniform and/or non-uniform doses and organ-specific absorbed doses [10].

The aim of this study was, therefore, to reassess the biokinetics in infants undergoing ^99m^Tc-MAG3 renal scans based on an image-based retrospective quantification and to derive organ absorbed doses as well as the associated risk for the related age group. The research comprised four steps: (1) phantom experiments for retrospective image quantification by depth- and size-dependent attenuation correction, (2) Estimation of patient-specific time-integrated activity coefficients (TIACs), (3) Calculation of the absorbed and effective doses, and (4) Risk assessment.

## Methods

### Patient data and measurement protocol

For this retrospective study, data from 20 consecutive patients with scintigraphy normal excretion with good wash-out were analyzed. Referral criteria for ^99m^Tc-MAG3 scintigraphy included sonographic suspicion of either urinary tract dilation or obstructive uropathy. The preparation of the patients (incl. oral hydration with 10 ml/kg or by breastfeeding 30 min prior to injection) was performed according to the EANM guidelines for standard and diuretic renograms in children [2]. Furosemide (1 mg/kg i.v. in infants, 0.5 mg/kg in children above the age of 1 year) was injected following a F+20 protocol in all but two patients who showed almost complete tracer excretion 20 min after ^99m^Tc-MAG3 administration. For a radiation-related absorbed dose and risk analysis, the patients were separated in two groups based on their age: 17 *newborns* (1.6–12.0 months) and 3 *1-year-olds* (13.0–20.0 months). As this study only included retrospectively analyzed data acquired within the clinical routine, our local ethics committee waived the need for further approval.

At our institution, ^99m^Tc-MAG3 scans are typically performed on a single-head gamma camera (E.Cam Signature, Siemens Healthcare) equipped with a low-energy high-resolution (LEHR) collimator. The injected activities are patient-specifically calculated based on the Pediatric Dosage Card 2014 of the European Association of Nuclear Medicine [4]. The study protocol is a planar dynamic acquisition of 132 images centered at the patients’ kidneys, started at the bolus injection, and lasting 35 min. The dynamic data are distributed in three phases (phase I: 40 images acquired over 1 min; phase II: 40 images acquired over 4 min; phase III: 52 images acquired over 30 min). The individual sizes and depths of each patient’s organs were taken from previously acquired ultrasound data.

### Determination of a depth- and size-dependent attenuation correction function

**Fig. 1:**
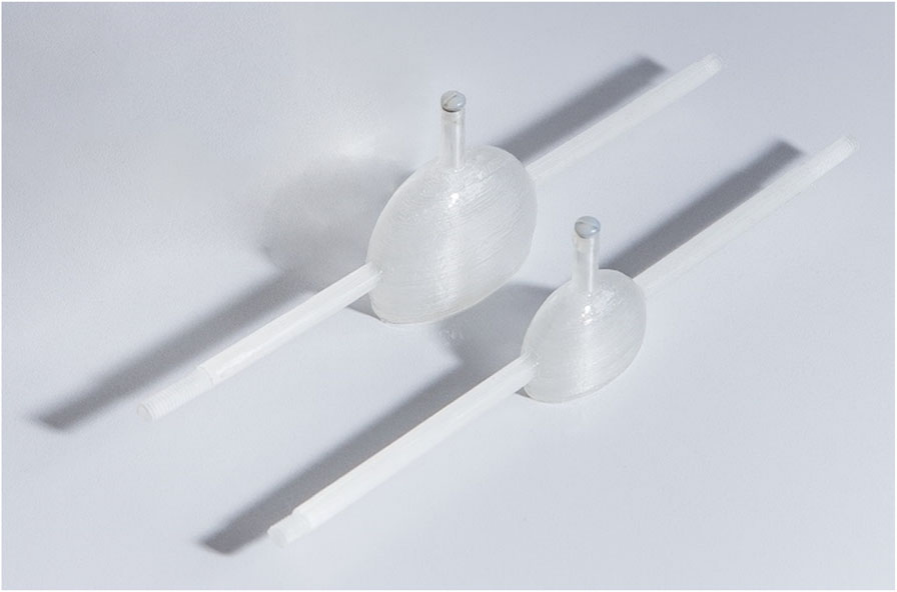
3D-printed 1-year-old and newborn kidney phantoms [12]

**Fig. 2:**
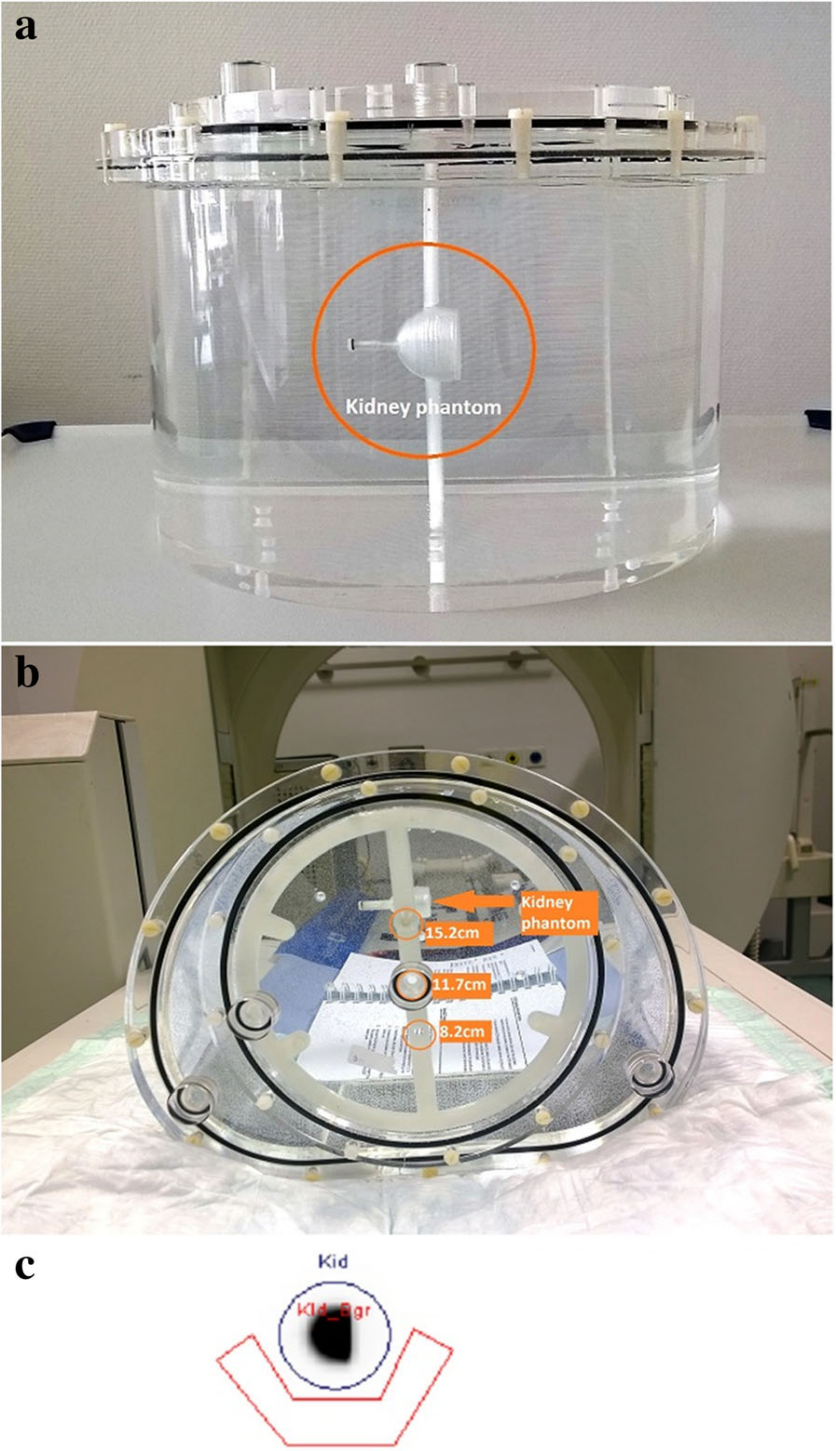
Phantom experiment. **a** Kidney insert (newborn) mounted on the torso-phantom. **b** Kidney insert mounted on the torso-phantom using the manufactured attachment system with three different depth positions: 8.2 cm, 11.7 cm, and 15.2 cm. **c** Exemplary ROI positioning for the phantom (*dark blue*) and the background (*red*)

To estimate the patient-specific organ activities, a retrospective calibration was performed on the same gamma camera that had been used for the patient acquisitions (E.Cam Signature, Siemens Healthcare). The goal was to derive a calibration factor by taking into account the sizes and depths of the individual kidneys. To simulate different kidney sizes, two one-compartment kidney phantoms designed according to MIRD pamphlet 19 [11] and fabricated with a 3D printer as described by Tran-Gia et al. [12] (Fig. 1) were used (newborns: 8.6 mL, 1-year-old: 23.4 mL). Both kidneys were filled with ^99m^Tc-solutions (newborn: 1.10 MBq/mL, 1-year-old: 0.98 MBq/mL). To simulate different kidney depths inside a patient, the phantoms were mounted in a body phantom (NEMA-NU2–2012, PTW-Freiburg) using a 3D-printed, depth-adjustable attachment system (distances of 8.2, 11.7, and 15.2 cm from the patient bed) which is also presented in [12] (Fig. 2a, b). After filling the phantom with water, static planar images (duration 600 s) were acquired for each depth position and kidney insert. Besides the acquisitions of the kidney phantom placed inside of the torso phantom, another acquisition was acquired with the kidney phantom placed directly on the patient bed to simulate a depth of 0 cm (i.e., approximately zero attenuation).

`

All post processing was performed with vendor-specific software (E.Soft, Siemens Healthcare). For each measurement, regions-of-interest (ROIs) were drawn around the phantom insert and in the background (Fig. 2c). To estimate the scatter contribution of the phantom as accurately as possible, a background subtraction was performed prior to any further calculations. As the background and phantom ROIs were different in size (Area_Phantom_ ≠ Area_Bgr_), a ROI normalization had to be applied to the counts in the background ROI (Counts_Bgr_):

1 $$ {\mathrm{Counts}}_{\mathrm{Bgr}\to \mathrm{Phantom}}(d)={\mathrm{Counts}}_{\mathrm{Bgr}}(d)\bullet \frac{{\mathrm{Area}}_{\mathrm{Phantom}}}{\ {\mathrm{Area}}_{\mathrm{Bgr}}} $$

Here, the parameter *d* represents the depth of the phantom (distance kidney ↔ patient bed). Based on the background counts normalized to the size of the phantom ROI (Counts_Bgr → Phantom_), a depth-dependent calibration factor cf_volume_ (unit: cps/MBq or counts-per-second-per-MBq) was calculated as:

2 $$ {\mathrm{cf}}_{\mathrm{volume}}(d)=\frac{{\mathrm{Counts}}_{\mathrm{Phantom}}(d)-{\mathrm{Counts}}_{\mathrm{Bgr}\to \mathrm{Phantom}}(d)}{A\ast \Delta  t} $$

where *A* represents the decay-corrected activity, and ∆*t* stands for the total acquisition duration.

Next, the depth-dependent calibration factors cf_volume_(*d*) were divided by the calibration factor at depth zero cf_volume_(*d*_0_) to obtain a unitless attenuation factor for a kidney-shaped organ of either age group (newborns and 1-year-olds):

3 $$ \mathrm{Attenuation}\ {\mathrm{Correction}\ \mathrm{Factor}}_{\mathrm{volume}}\ (d)=\frac{{\mathrm{cf}}_{\mathrm{volume}}(d)}{{\mathrm{cf}}_{\mathrm{volume}}\left({d}_0\right)} $$

To enable an attenuation correction for patient-specific organ depths, a depth-dependent attenuation correction function was approximated by a second-degree polynomial curve, which was separately fitted to the newborn and the 1-year-old data. Figure 4 shows these attenuation correction factors as a function of the depth *d* for newborns (blue) and 1-year-olds (orange). The calibration factor for any depth can be calculated based on these curves by inserting the patient-specific organ depth (Additional file 1: Table S1) into the fitted equations shown in the graphic.

### Determination of time-activity curves in the patients

**Fig. 3:**
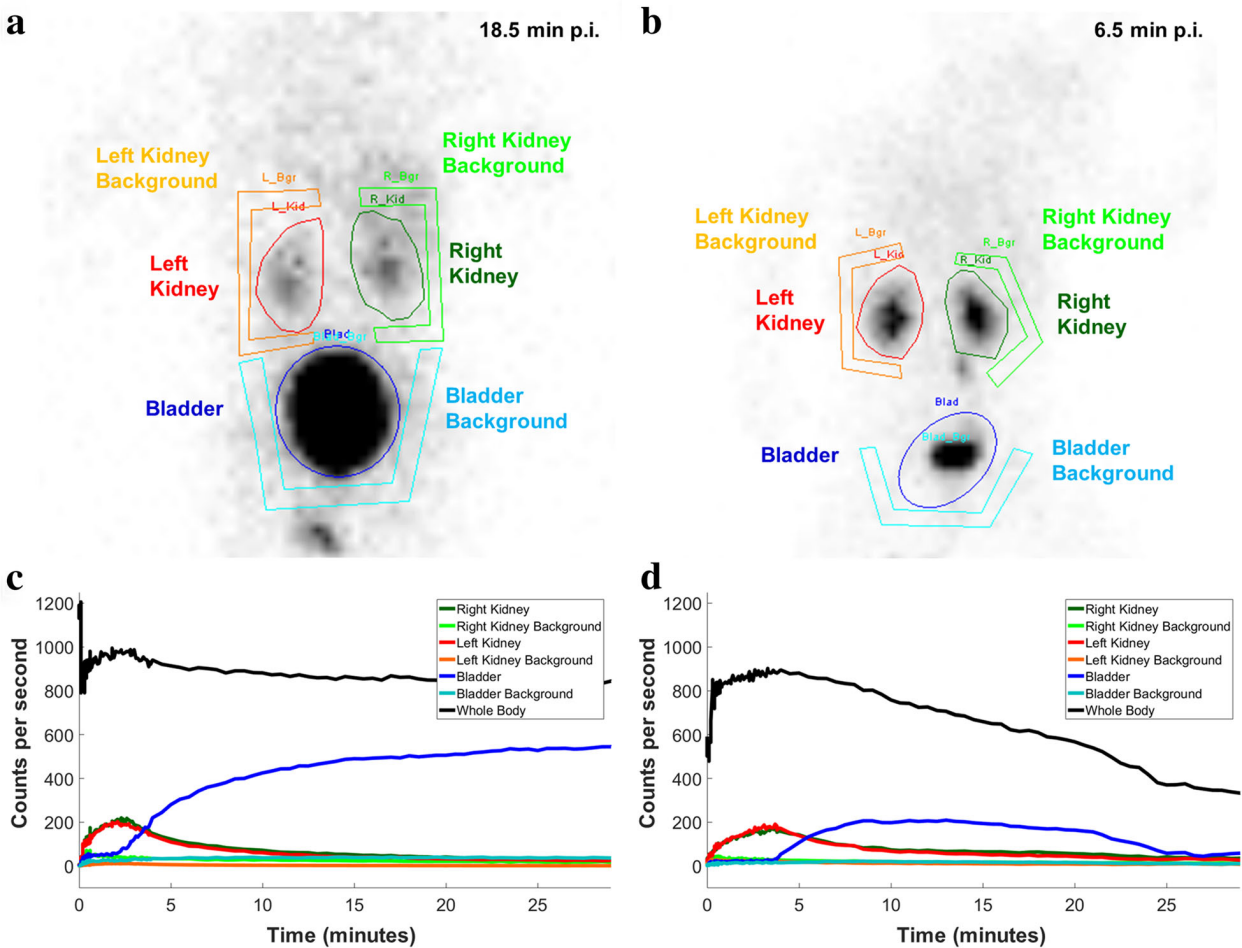
ROI analysis of patients P20 (left, 20-month-old female) and P10 (right, 5-month-old female). **a**, **b** ROIs and background in kidneys (*red*: left kidney; *green*: right kidney) and bladder (*blue*) for multiple time points. The whole-body ROI covers the entire field-of-view and is not depicted. **c**, **d** Number of counts as a function of time for all ROIs

Another ROI analysis was performed to obtain the time-activity curves for different organs from the patient data. The ROIs were drawn around the kidneys and the bladder, with an additional ROI placed beside each organ to estimate the background (Fig. 3). The whole-body ROI (WB) covered the entire field-of-view. After background correction (subtraction of the counts in the background ROI normalized to the organ area from the organ ROI as described by Eqs. 1 and 2), the result was a temporal course of the counts in all examined organs.

**Fig. 4:**
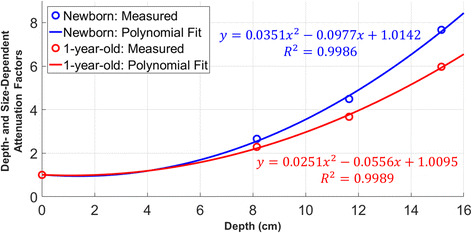
Depth-dependent attenuation correction factors obtained in the phantom experiment. *Blue*: newborns. *Red*:1-year-olds

The conversion from counts to activity was performed based on pre-determined ultrasound-based patient data (kidney and bladder volumes, kidney depth): First, the kidney depth was inserted into both attenuation correction functions (Fig. 4) to obtain depth-corrected attenuation factors for the newborn and 1-year-old volumes. Subsequently, the calibration factor for the patient-specific kidney volume was linearly interpolated based on these volume-cf pairs (Fig. 4). Finally, kidney time-activity curves were obtained by dividing the number of counts in each temporal frame by the resulting factor and the acquisition time.

The bladder time-activity curves were determined similarly, with the exception that no depth could be extracted from the ultrasound data. Instead, a depth of 5 cm was used for all bladders. The assumption that the kidney geometry is comparable to the approximately spherical geometry of the bladder is based on a study by Tran-Gia et al., where only a negligible difference occurred between the MIRD-based kidneys and a spherical model of similar volume [12].

The whole-body time-activity curves were obtained under the assumption that the total number of counts corresponded to the total administered activity. This typically holds for pediatric patients, as a large part of the body is included in the camera field-of-view. The coverage sufficiently represents the normalized whole-body biokinetics.

### Patient-specific time-integrated activity coefficients

The patient-specific time-integrated activity coefficients (TIACs) were obtained by a time integration of the organ-specific time-activity curves. During the renal scans, 132 images were acquired starting at the bolus injection over a period between 0 and 35 min. While the organ-specific time-activity curves were integrated up to the last time point for each patient, only physical decay was assumed after the last time point. A combination of a trapezoidal integration for time points before the curve maximum and a bi-exponential function after the maximum was applied to all time-activity curves. While the bi-exponential functions were fitted in OriginPro 2016 (ADDITIVE GmbH), the integrals were calculated using Microsoft Office 365 Excel version 2016 (Microsoft Corporation).

### Absorbed dose calculation

The absorbed dose coefficients (mGy/MBq) to the organs and the effective dose coefficients (mSv/MBq) for each patient were assessed by means of the newborn and the 1-year-old mathematical phantom provided by OLINDA/EXM [13].

### Risk estimation

The risk estimation was performed individually for all 20 pediatric patients with RadRAT, published by the US National Cancer Institute. The following input data were used: gender; age; population group (U.S. 2000–2005); exposure year; organs like brain, breast (females), colon, gallbladder, liver, lungs, ovaries (females), kidneys, pancreas, red bone marrow, stomach, thyroid, urinary bladder, uterus (females); exposure rate (acute); dose distribution type (fixed value); organ-specific absorbed doses [10]. The result was the percentage risk in 100,000 persons for the development of stochastic radiation-induced effects for “lifetime attributable risk” and “future risk”. The lifetime attributable risk (LAR) estimates the probability of cancer development and death by an individual arising from radiation exposure. The future risk is defined as the risk estimated for an individual from the present time until the end of the expected lifetime for developing cancer [6, 10].

Despite a high uncertainty in the individual risk estimation (90%), this information might assist in the establishment of more accurate recommendations for this high-risk group of pediatric patients for keeping the balance between sufficient imaging quality at the lowest possible patient radiation exposure [14].

## Results

All values will be given as mean plus/minus one standard deviation except otherwise specified.

### Demographic data of the patient group

The Additional file 1: Table S2 shows the demographic data of the 20 patients (14 males; 6 females) classified into the two age groups (newborns and 1-year-olds). Ages are 1.6–20.0 months (mean 7.0 ± 4.6 months). Weight is 5–12 kg (mean 7.8 ± 1.9 kg). Body size is 60–89 cm (mean: 69.5 ± 7.8 cm). Injected activity is 12–24 MBq (mean 17.9 ± 2.6 MBq). Four patients had injected activities between 12 and 15 MBq, 14 patients between 16 and 20 MBq and 2 patients between 21 and 24 MBq. On average, the activities administered to our patients were 22% lower than the recommended injected activity values from EANM Dosage Card 2014 [4].

### Depth- and size-dependent attenuation correction functions

The depth- and size-dependent attenuation correction curves are shown in Fig. 4. Separate second-degree polynomials were fitted separately for the newborn and the 1-year-old kidney for distances of 0, 8.2, 11.7, and 15.2 cm from the patient bed. As expected, the attenuation increases with the distance. While the attenuation is comparable for small depths < 5 cm (no difference for 0 cm), the attenuation of the newborn kidney phantom is higher than that of the 1-year-old phantom for larger depths (differences of 14% for 8.2 cm, 18% for 11.7 cm, and 22% for 15.2 cm).

### Patient-specific time-integrated activity coefficients

The time-integrated activity coefficient (TIAC) values of the patient analysis are given in Table 1.

**Table 1:** Organ-specific time-integrated activity coefficients (TIACs) in hours for all patients (classified into age groups)

Age group	Patient	Gender	TIAC (h)		
			Kidneys	Bladder	Wholebody
Newborns (1.6–11.0 months; 13 males (M), 4 females (F))	P01	M	0.17	0.42	1.32
	P02	M	0.11	0.30	0.70
	P03	M	0.16	1.30	0.71
	P04	F	0.06	0.21	0.59
	P05	M	0.07	0.31	3.88
	P06	M	0.07	0.38	3.57
	P07	M	0.07	2.94	0.61
	P08	F	0.08	2.92	0.75
	P09	M	0.03	1.97	0.35
	P10	F	0.05	0.58	0.43
	P11	M	0.07	0.21	1.55
	P12	M	0.29	1.39	0.57
	P13	M	0.05	0.06	0.69
	P14	M	0.15	1.34	3.66
	P15	M	0.09	0.05	0.35
	P16	F	0.44	0.93	3.12
	P17	M	0.07	0.14	3.35
	Mean ± SD		0.11 ± 0.09	0.91 ± 0.92	1.54 ± 1.32
1-year-olds (13.0–20.0 months; 1 male (M), 2 females (F))	P18	M	0.05	1.11	0.17
	P19	F	0.10	1.32	0.43
	P20	F	0.03	2.99	0.31
	Mean ± SD		0.06 ± 0.03	1.81 ± 0.84	0.31 ± 0.11
All	Mean ± SD		0.11 ± 0.09	1.04 ± 0.96	1.36 ± 1.29
1-year-olds—ICRP 128 [15]			0.065	1.6	0.23

Comparing the patient age groups, the mean TIAC values for the newborn group were 47% higher for the kidneys, 50% lower for the bladder, and 80% higher for the whole-body. Although none of the patients had severe kidney function impairments, large inter-patient variations were observed. However, according to one-way ANOVA tests comparing the TIAC values between the newborn and the 1-year-old groups (*p* value 0.07), there was no significant difference (*p* > 0.05). In comparison with the ICRP 128 values for the 1-year-old group with normal renal function (kidneys 0.065 h; bladder 1.6 h; whole-body 0.23 h) [15], the differences of the mean TIAC values were 38% higher for the kidneys, 35% lower for the bladder, and 83% higher for the whole-body.

### Dose calculation

Additional file 1: Table S3 shows the mean values of the organ-specific absorbed dose coefficients. For all patients, the kidneys absorbed dose coefficient values were between 0.004 and 0.131 mGy/MBq and the bladder absorbed dose coefficients were 0.01–0.93 mGy/MBq. The effective dose coefficients ranged from 0.001 to 0.063 mSv/MBq.

Table 2 shows the dose results clustered per age group. Comparing the two age groups, the newborns showed 66% higher absorbed doses for the kidneys. The 1-year-old group showed 6% higher bladder absorbed doses. The effective dose results were 24% higher for the 1-year-old group.

**Table 2:** Mean absorbed doses and effective doses for both age groups

Age group	Absorbed dose (mGy)		Effective dose (mSv)
	Kidney	Bladder	
Newborns	0.69 ± 0.55	4.79 ± 4.70	0.36 ± 0.25
1-year-olds	0.23 ± 0.15	5.08 ± 1.50	0.48 ± 0.23
All	0.62 ± 0.53	4.84 ± 4.37	0.38 ± 0.25

In contrast to adults, excretion cannot be controlled or contained by newborns and toddlers. Therefore, it is possible to observe the influence of bladder voiding on the dosimetry. The patients who had bladder voiding before the last image (i.e., within ~ 30 min after the injection) showed lower absorbed dose values. The mean organ absorbed doses for the 13 patients with voiding were 0.5 ± 0.2 mGy for the kidneys, 3.3 ± 2.6 mGy for the bladder, and 0.1 ± 0.1 mGy for the whole-body. As expected, the mean organ absorbed doses for the 7 patients without voiding were 47% higher in the kidneys (0.9 ± 0.8 mGy), 57% higher in the bladder (7.7 ± 5.5 mGy), and 35% higher for the whole-body (0.2 ± 0.1 mGy).

### Risk estimation

The results of the excess lifetime risk estimation are given in Table 3. The mean excess lifetime risk, as well as the lower and upper bounds (limits) of the respective confidence intervals (CI) for the risk probability of all patients, classified per age and gender group, are listed in Table 3. The group of *newborn* patients has a mean risk value of 16.8 per 100,000 persons to develop cancer from radiation exposure, which is about 12% higher compared to that of the *1-year-old* group (14.7 per 100,000). Comparing the mean excess lifetime risk values between different gender groups (Table 3), the female patients have a 29% higher risk than male patients. Related to the excess lifetime risk per cancer site (Additional file 1: Table S4), the main critical organs featuring higher risk values for the underlying patient group are the bladder, colon, and kidneys.

**Table 3:** Age- and gender-dependent mean excess lifetime risk (chances in 100,000 persons)

Age group	Newborns (1.6–11.0 months)	1-year-olds (13.0–20.0 months)	Males (14 patients)	Females (6 patients)
Excess lifetime risk (in 100,000)	16.8 ± 13.5	14.7 ± 4.3	14.7 ± 12.7	20.7 ± 10.4
Lower bound	6.8	5.5	5.7	8.6
Upper bound	33.1	29.2	29.8	39.2
Age (months)	5.4 ± 2.4	15.7 ± 3.1	6.0 ± 3.1	9.3 ± 6.0

RadRAT—Lifetime risk of developing cancer of the exposed organs with 90% uncertainty range [10]

**Fig. 5:**
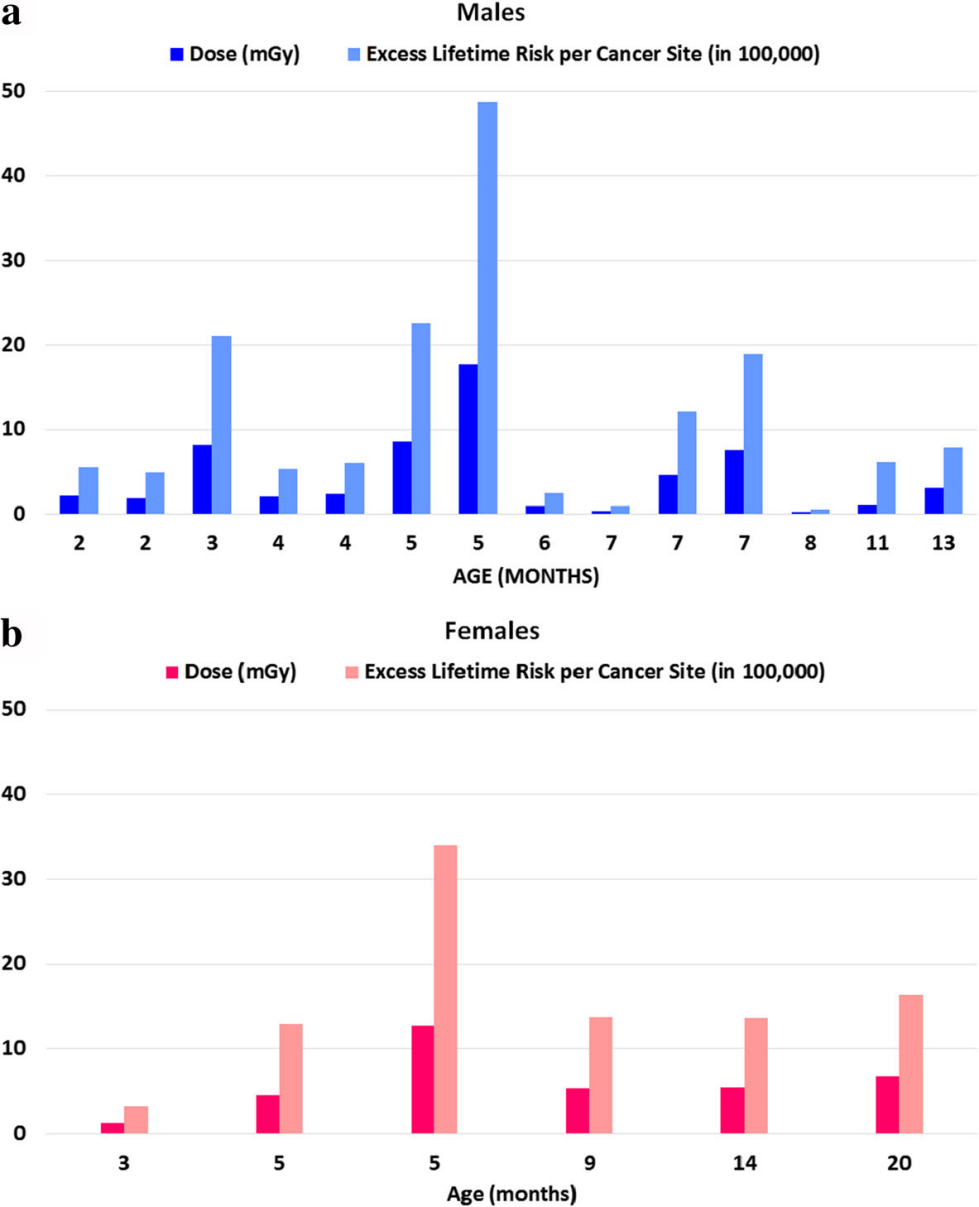
Absorbed doses to the bladder (mGy) and excess lifetime risk (chances in 100,000 persons) as a function of age (months). **a** Male group with 14 patients aged between 2 and 13 months. **b** Female group with 6 patients aged between 3 and 20 months

Figure 5 shows a comparison between the individual absorbed doses to the bladder and excess lifetime risk for the 20 patients. As expected, increased organ absorbed doses lead to a higher risk, independently of the age.

## Discussion

This retrospective study in infants with normal kidney function undergoing ^99m^Tc-MAG3 is the first comprehensive study on biokinetics, dosimetry, and radiation-related risk in a larger group of patients. For retrospective image quantification, age-specific 3D-printed phantoms were manufactured and calibration measurements were performed.

For this study, we chose, for a direct comparison to the published data by Stabin et al. [16] and to the ICRP 128 data [15], to use the Cristy-Eckerman stylized phantoms provided by OLIDNA/EXM [13]. In addition, the effective doses provided by ICRP 128 are still calculated with the ICRP 60 tissue weighting factors [9]. Although new hybrid phantoms for pediatric patients have been developed by the University of Florida group and have been applied by Sgouros et al. in their study on DSMA absorbed doses [14], we believe that, for a retrospective organ dose assessment as performed in this study in a limited number of source organs (kidneys, bladder, whole-body), the accuracy of the dose calculation with OLINDA/EXM is sufficient as a basis for a risk estimate.

Compared to the pediatric patients’ ^99m^Tc-MAG3 data presented by Stabin et al. in [16], the absorbed dose coefficients observed in our study are lower for the newborns and higher for the 1-year-old patients. The kidney absorbed dose coefficients were 17% lower for the newborns and 25% higher for the 1-year-old group, and 22% lower for newborns and 76% higher for 1-year-olds in the bladder. Lastly, the remainder dose coefficient was 7% lower for newborns and 63% higher for 1-year-olds. This can be related to the difference of the number of patients (our study has 20 pediatric patients and Stabin et al. study has two pediatric patients in the age range considered) [16].

In an intravenous urography (IVU) study, Almen et al. showed an average absorbed dose per exposure of 0.68 mGy (range 0.48 to 1.10 mGy) for pediatric patients aged between 0 and 1 year [17]. In comparison, the ^99m^Tc-MAG3 scans presented in this study resulted in a 9% lower kidney absorbed dose of 0.62 mGy average over 20 patients (range 0.10 to 2.62 mGy).

Overall, the mean effective dose per patient was less than 1 mSv, showing that the recommendations for administered activities based on the patient weight (EANM Pediatric Dosage Card 2014) keeps the absorbed doses low for pediatric patients undergoing renal ^99m^Tc-MAG3 examinations [4].

The patients’ mean excess lifetime risk was 16.5 per 100,000 people (lower boundary 6.6; upper boundary 32.6). Gender-wise, the male patient group showed a mean excess lifetime risk value of 14.7 per 100,000 persons compared to 20.7 per 100,000 for the female group (Table 3). These risk values are the lifetime overall risk for developing cancer.

Please find the corrected second paragraph (with the corrected values denoted in bolditalics) here:

“Based on information from the US National Cancer Institute’s Surveillance Epidemiology and End Results (SEER) of the American Cancer Society (database: 2010 to 2012), the risk for developing cancer is 42% in the males, and 38% in females [18]. The lifetime overall risk in male population is ***2,864*** times higher than the mean excess lifetime risk of our patients. Compared to the female population, the mean excess lifetime risk of our patients is approximately ***1,817*** times lower for all cancer types [18]. Similar results are shown for a comparison with the risk database (2012) from the Robert Koch Institute’s (RKI) German Centre for Cancer Registry Data [19]. In Germany, the male population showed a lifetime overall risk of 50% for developing cancer [19], which is about ***3,440*** times higher than the mean excess risk for our male patients. The female population has a lifetime overall risk of 43% for developing cancer, which is approximately ***2,084*** times higher than the excess risk for our female patients [19]. According to these comparisons, the overall additional risk for our patient group can be considered as very low.”

As expected, the excess lifetime risk of our pediatric patient group in comparison with adults undergoing the same exposure was higher for both genders. As an example, we estimated the excess lifetime risk of a 30-year-old adult by separately inserting the organ doses of male patient P18 and female patient P20 at the same exposure year in the RadRAT [10]. While the adult male showed a 61.2% lower risk, the adult female showed a 60.8% lower risk.

Based on a cohort of atomic bomb survivors, the study of Ozasa et al. [20] showed that the risk of a higher mortality caused by late effects of radiation exposure is increased during the lifespan. The rates of cancer deaths increased in proportion to age and dose of radiation [20]. In this cohort, the individuals who were exposed at younger ages presented a higher risk for different cancer sites [20]. In contrast, the risk decreases for those who were exposed at older ages [20–22].

Additional file 1: Table S4 presents the mean excess lifetime risk values clustered per cancer site for all patients. Except for the bladder, all other included organs show a maximum risk of 1 per 100,000 persons. The critical organs (highest risk values) were the bladder, colon, thyroid, lungs, kidneys, and bone marrow. In comparison, Ozasa et al. [20] presented similar results: besides the organs stated above, the breast (female), esophagus, gall bladder, and liver were reported as organs with the highest excess risk per cancer site. Conversely, the rectum, uterus (female), prostate (male), and kidneys (parenchyma) presented no significant excess risk [20].

The highest mean organ absorbed doses were observed in the bladder with values above 4 mGy. Thus, the estimated risk was higher for all ages and genders (Fig. 5). For male patients, the bladder absorbed doses were 28% lower than for female patients with the risk accordingly reduced by 25%.

And find here the corrected seventh paragraph (with the corrected values denoted in bolditalics): 

“Compared to our patient risk data, the lifetime overall risk for both genders of the general population for developing bladder cancer is above the mean excess lifetime risk, with values of approximately ***329*** (SEER) and ***214*** (RKI) times higher for males and ***73*** (SEER) and ***51*** (RKI) times higher for females [18, 19]. Bladder voiding influenced the risk, in comparison to the patient group without bladder voiding during the examination, the mean excess lifetime risk values of the patient group with voiding was 58% lower.”

Our results show a tendency towards higher excess lifetime risks for female patients compared to males (Table 3) and gender-specific distinctions when comparing the organs’ dose-risks between both genders (Additional file 1: Table S4) [6, 7, 20]. As an example, the excess lifetime risk values for the kidneys were higher for males than for females [20].

A one-way ANOVA test was performed to examine significant differences between the patient groups. The input data were the results for absorbed doses (mGy), excess lifetime risk (chances in 100,000), and excess lifetime risk per cancer site (chances in 100,000) for both age groups (newborns and 1-year-old groups) and both genders (male and female). According to the tests, no significant differences were found (*p* > 0.05). The *p* values for the age groups were 0.2 for kidneys absorbed dose (mGy), 0.9 for bladder absorbed dose (mGy), and 0.8 for excess lifetime risk (chances in 100,000). The *p* value for the gender groups was 0.4 for excess lifetime risk (chances in 100,000).

There are some shortcomings concerning the study; however, as it is a retrospective study with images taken at suboptimal time points for dosimetry, the doses reported might be overestimated due to the approximation of a physical decay after the last time point. The error associated with the calibration and the subsequent patient-specific correction adds to the uncertainty of the dose assessment. In this age group, however, the variability concerning morphology is rather low. For an estimate of the effective doses according to ICRP 103, the risk factors could not be applied as the data of the underlying voxel-based ICRP phantom are yet to be published [5].

Nevertheless, a risk-adapted, TIAC-based approach applied for organ-specific absorbed dose calculations, instead of reporting effective dose values obtained by multiplying the administered activities with constant values taken from the ICRP tables such as ICRP 128 [15], might lead to improvements of future recommendations for pediatric dosages in nuclear medicine diagnostics.

## Conclusion

In this study, we retrospectively derived new data on biokinetics and dosimetry for infants with normal kidney function after undergoing renal ^99m^Tc-MAG3 scans. In addition, we analyzed the associated age- and gender-specific excess lifetime risk due to ionizing radiation. The absorbed and effective doses were low when using the EANM pediatric dosage card for calculating the injected activities. The radiation-associated stochastic risk increases with the organ doses taking age- and gender-specific influences into account. In comparison with adults, the pediatric patient data show a slightly higher radiation-related risk (excess lifetime risk) for the same absorbed doses. Overall, however, the lifetime radiation risk associated with the ^99m^Tc-MAG3 scans is very low when compared to the general population’s risk for developing cancer.

## Additional file

Additional file 1: Table S1. Patient-specific organ sizes classified per age groups (newborns and 1-year-olds). **Table S2.** Demographics clustered by age groups with patients’ information of age, gender, weight, body size, and injected activity. **Table S3.** The values for Patient organ-specific mean absorbed dose coefficients. **Table S4.** Mean organ-specific absorbed doses and respective estimated excess lifetime risk per cancer site (chances in 100,000 persons) for newborns (1.6–11.0 months) and 1-year-olds (13.0–20.0 months) clustered per gender. (PDF 1213 kb)
