# Correction to: Biokinetics, dosimetry and radiation risk in infants after ^99m^Tc-MAG3 scans

**DOI:** 10.1186/s13550-021-00836-0

**Published:** 2021-10-11

**Authors:** J. Soares Machado, J. Tran-Gia, S. Schlögl, A. K. Buck, M. Lassmann

**Affiliations:** Department of Nuclear Medicine, University of Würzburg, Oberdürrbacher Str. 6, 97080 Würzburg, Germany

## Correction to: EJNMMI Research (2018) 8:10. https://doi.org/10.1186/s13550-017-0356-2

Following publication of the original article [1], the authors identified several calculation errors in the second and seventh paragraphs of the discussion section.

Please find the corrected second paragraph (with the corrected values denoted in italics) below:

“Based on information from the US National Cancer Institute’s Surveillance Epidemiology and End Results (SEER) of the American Cancer Society (database: 2010 to 2012), the risk for developing cancer is 42% in the males and 38% in females [2]. The lifetime overall risk in male population is *2864* times higher than the mean excess lifetime risk of our patients. Compared to the female population, the mean excess lifetime risk of our patients is approximately *1817* times lower for all cancer types [2]. Similar results are shown for a comparison with the risk database (2012) from the Robert Koch Institute’s (RKI) German Centre for Cancer Registry Data [3]. In Germany, the male population showed a lifetime overall risk of 50% for developing cancer [3], which is about *3440* times higher than the mean excess risk for our male patients. The female population has a lifetime overall risk of 43% for developing cancer, which is approximately *2084* times higher than the excess risk for our female patients [3]. According to these comparisons, the overall additional risk for our patient group can be considered as very low.”

And please find here the corrected seventh paragraph (with the corrected values denoted in italics):

“Compared to our patient risk data, the lifetime overall risk for both genders of the general population for developing bladder cancer is above the mean excess lifetime risk, with values of approximately *329* (SEER) and *214* (RKI) times higher for males and *73* (SEER) and *51* (RKI) times higher for females [2, 3]. Bladder voiding influenced the risk, in comparison to the patient group without bladder voiding during the examination, the mean excess lifetime risk values of the patient group with voiding was 58% lower.”

The authors apologize for any inconvenience caused.
