# Supplementary material for: Biokinetics, dosimetry, and radiation risk in infants after 99mTc-MAG3 scans
Source: EJNMMI Res. 2018 Feb 2;8:10. doi: 10.1186/s13550-017-0356-2 (PMC5796928; doi:10.1186/s13550-017-0356-2)
Supplement: Supplementary file 1 — Patient-specific organ sizes classified per age groups (newborns and 1-year-olds). Table S2. Demographics clustered by age groups with patients’ information of age, gender, weight, body size, and injected activity. Table S3. The values for Patient organ-specific mean absorbed dose coefficients. Table S4. Mean organ-specific absorbed doses and respective estimated excess lifetime risk per cancer site (chances in 100,000 persons) for newborns (1.6–11.0 months) and 1-year-olds (13.0–20.0 months) clustered per gender. (PDF 1213 kb) [file 13550_2017_356_MOESM1_ESM.pdf]

Biokinetics, Dosimetry and Radiation Risk in Infants after ^{99m}Tc-MAG3 Scans

J. Soares Machado, J. Tran-Gia, S. Schlögl, A. K. Buck, M. Lassmann

Department of Nuclear Medicine, University of Würzburg, Würzburg, Germany

Short title: Dosimetry and Risk in Infants after ^{99m}Tc-MAG3 Scans

First and Corresponding author:

Jéssica Soares Machado

Department of Nuclear Medicine

University Hospital Würzburg

Oberdürrbacher Str. 6

97080 Würzburg

Germany

Phone: +49 - 931-20144420

Email : E_Soares_J@ukw.de

Autours' Email :

J. Tran-Gia: Tran_J@ukw.de

S. Schlögl: Schloegl_s@ukw.de

A. K. Buck: buck_a@ukw.de

M. Lassmann: Lassmann_m@ukw.de

Key words:

Pediatric patients, Dosimetry, ^{99m}Tc-MAG3, Biokinetics, Absorbed dose, Risk assessment.

Additional files

Table S1. Patients-specific organ sizes classified per age groups (newborns and 1-year-old).

Age group	Patient	Anatomical Data				
		**Right Kidney**		**Left Kidney**		**Bladder**
		Depth	Volume	Depth	Volume	Volume
		(cm)	(ml)	(cm)	(ml)	(ml)
Newborns	P1	1.6	17.0	1.7	20.0	34.0
	P2	2.5	28.1	2.5	24.3	35.0
	P3	2.6	18.0	2.0	15.6	11.6
	P4	2.3	19.1	2.2	14.1	37.5
	P5	2.1	19.0	2.6	11.4	40.0
	P6	2.3	24.0	2.3	19.0	11.0
	P7	2.6	18.0	2.5	18.0	42.5
	P8	2.3	29.1	2.3	19.8	42.5
	P9	2.7	18.0	2.4	17.0	42.5
	P10	2.5	16.7	2.4	15.0	5.0
	P11	2.7	28.1	2.5	24.3	45.0
	P12	2.7	19.0	2.1	20.0	6.0
	P13	2.4	14.0	2.7	20.0	51.0
	P14	2.4	31.0	2.3	29.0	19.0
	P15	2.8	32.0	2.8	25.0	30.0
	P16	3.2	19.3	2.7	25.6	52.5
	P17	3.0	25.0	2.2	23.0	19.0
**MEAN**		**2.5±0.4**	**22.1±5.5**	**2.4±0.3**	**20.1±4.5**	**30.8±15.3**
1-year-old	P18	1.7	60.0	1.8	46.0	92.5
	P19	1.8	20.0	2.6	21.0	60.0
	P20	2.2	30.0	2.8	35.0	65.0
**MEAN**		**1.9±0.2**	**36.7±17.0**	**2.4±0.4**	**34.0±10.2**	**72.5±14.3**
**ALL**		**2.4±0.4**	**24.3±9.8**	**2.4±0.3**	**22.2±7.6**	**37.1±21.2**

The individual sizes and depths of each patient's organs were taken from previously acquired ultrasound data. As the individual depth (cm) information for bladder could not be extracted from the ultrasound exams, it was applied 5.0 cm for all patients.

Table S2. Demographics clustered by age groups with patients' information of age, gender, weight, body size and injected activity.

Age group	Patient	Age	Gender	Weight	Body Size	Injected Activity
		**(month)**		**(kg)**	**(cm)**	**(MBq)**
Newborns *(1.6-11.0 months; males:13; females: 4)*	P1	1.6	M	5.0	62	12
	P2	2.0	M	5.0	60	15
	P3	3.0	M	6.3	62	18
	P4	3.0	F	5.0	59	14
	P5	4.0	M	6.0	63	18
	P6	4.0	M	7.0	65	15
	P7	5.0	M	7.0	67	19
	P8	5.0	F	8.0	72	16
	P9	5.0	M	8.0	66	16
	P10	5.0	F	7.0	66	19
	P11	6.0	M	9.5	72	20
	P12	7.0	M	7.0	68	17
	P13	7.0	M	8.0	65	19
	P14	7.0	M	10.0	76	18
	P15	8.0	M	9.0	70	19
	P16	9.0	F	8.0	72	20
	P17	11.0	M	8.0	74	21
**MEAN**		**5.4±2.4**		**7.3±1.5**	**67.0±4.9**	**17.4±2.4**
1-year-old *(13.0-20.0 months; males:1; females: 2)*	P18	13	M	11.0	87	20
	P19	14	F	9.0	74	18
	P20	20	F	12.0	89	24
**MEAN**		**15.7±3.1**		**10.7±1.2**	**83.3±6.6**	**20.7±2.5**
**All**		**7.0±4.6**		**7.8±1.9**	**69.5±7.8**	**17.9±2.6**

Table S3. The values for patient organ-specific mean absorbed dose coefficients

Age Group	Absorbed Dose Coefficient (mGy/mBq)		Effective Dose Coefficient (mSv/MBq)
	**Kidney**	**Bladder**	
**Newborns**	0.04±0.03	0.27±0.24	0.02±0.02
**1y**	0.01±0.01	0.25±0.06	0.02±0.00
**All**	**0.04±0.03**	**0.27±0.24**	**0.02±0.02**

Table S4. Mean organ-specific absorbed doses and respective estimated excess lifetime risk per cancer site (chances in 100,000 persons) for newborns (1.6-11.0 months) and 1-year-old (13.0-20.0 months) clustered per gender.

Organs	MALE *(14 Patients)*		FEMALE *(6 Patients)*	
	**DOSE** (mGy)	**RISK** (in 100,000)	**DOSE** (mGy)	**RISK** (in 100,000)
**Stomach Wall**	0.1±0.1	0.3±0.2	0.1±0.1	0.2±2.0
**Colon**	0.3±0.2	1.0±0.6	0.3±0.2	0.8±0.4
**Liver**	0.1±0.1	0.2±0.1	0.1±0.1	0.1±0.1
**Gallbladder Wall**	0.1±0.1	0.0±0.0	0.1±0.1	0.0±0.0
**Pancreas**	0.2±0.1	0.1±0.1	0.1±0.1	0.1±0.1
**Lungs**	0.1±0.1	0.4±0.3	0.1±0.1	0.6±0.7
**Urinary Bladder**	4.3±4.8	11.7±12.4	6.0±3.5	15.6±9.2
**Kidneys**	0.6±0.3	0.5±0.2	0.7±0.9	0.4±0.6
**Brain**	0.1±0.1	0.1±0.1	0.1±0.1	0.0±0.0
**Thyroid**	0.1±0.1	0.3±0.2	0.1±0.1	0.9±1.0
**Red Marrow**	0.1±0.1	0.4±0.4	0.1±0.1	0.3±0.3
**Ovaries**	-	-	0.3±0.2	0.3±0.2
**Breasts**	-	-	0.1±0.1	0.8±0.7
**Uterus**	-	-	0.6±0.3	0.4±0.2

RadRAT Tool - Lifetime Risk of developing Cancer of the Exposed organs with 90% uncertainty range[1].

References

1. De Gonzalez, A. B., Apostoaei, A. I., Veiga, L. H. S., Rajaraman, P., Thomas, B. A. et al. (2012). RadRAT: A Radiation Risk Assessment Tool for Lifetime Cancer Risk Projection. Journal of Radiological Protection : Official Journal of the Society for Radiological Protection, 32(3), 10.1088/0952–4746/32/3/205. http://doi.org/10.1088/0952-4746/32/3/205
